# High levels of thyroid hormones promote recurrence of Graves' disease via overexpression of B‐cell‐activating factor

**DOI:** 10.1002/jcla.24701

**Published:** 2022-09-13

**Authors:** Shu Liu, Jing‐Jing Miao, Xiao Zhou, Qi Sun, Xiao‐Ming Mao

**Affiliations:** ^1^ Department of Endocrinology, Nanjing First Hospital Nanjing Medical University Nanjing China

**Keywords:** abnormal differentiation of B cells, autoreactive antibody‐secreting B cells, CD11c, Graves' hyperthyroidism, triiodothyronine

## Abstract

**Background:**

Elevated thyroid hormone (TH) levels have been suggested to be associated with the pathological progression of Graves' disease (GD). However, direct evidence from clinical studies remains unclear.

**Methods:**

Peripheral blood samples were collected from patients with or without the recurrence of Graves' hyperthyroidism (GH) and healthy donors. Thyroid tissue samples were obtained from patients with benign thyroid nodules. To assess the differentiation of autoreactive B cells, the expression of B‐cell‐activating factor (BAFF) and the proportion of CD11c+/–IgG+/− subsets of B cells stimulated by high levels of triiodothyronine (T3) in vivo and in vitro were examined by ELISA, flow cytometry, western blotting, and qRT‐PCR.

**Results:**

Serum BAFF levels in patients with GD were significantly and positively correlated with FT3, FT4, and TRAb levels. Furthermore, the ratio of abnormally differentiated CD11c+ autoreactive B cells positively correlated with BAFF and TRAb. High levels of triiodothyronine (T3) induced BAFF overexpression in thyroid follicular cells and mononuclear cells of the normal thyroid in vitro, thereby promoting the differentiation of CD11c+IgG+ autoreactive secretory B cells (ASCs). However, the precise knockdown of BAFF expression significantly inhibited the abnormal differentiation of ASCs.

**Conclusion:**

The pathological progression of GD was prolonged and exacerbated by autoimmune positive feedback modulation caused by high TH levels. BAFF could be considered a potential target for localized thyroid immunosuppressive treatment of Graves' hyperthyroidism recurrence.

## Graphical abstract

In the present study, we found that the pathological progression of GD was prolonged and exacerbated by positive feedback regulation of abnormal differentiation of local autoimmune secretory B cells (ASCs) in the thyroid induced by high TH levels, in which BAFF plays a key bridging role and can be considered as a potential target for local thyroid immunosuppression in the treatment of Graves' hyperthyroidism relapse.

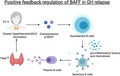

## 1 INTRODUCTION

Graves' disease (GD) is a common autoimmune disorder of the thyroid gland characterized by hyperthyroidism, in which the activation of B cells and production of thyrotropin receptor antibody (TRAb) continuously stimulates thyroid follicular cell (TFC) proliferation, synthesis, and secretion of thyroid hormones (THs). The hypothalamic–pituitary–thyroid (HPT) axis has been established as an integral acute stress‐responsive neuroendocrine system, and fluctuations in circulating TH levels in patients with GD are coordinated by this axis.^1^, ^2^

The THs mainly comprise triiodothyronine (T3) and tetraiodothyronine (T4), where T3 is the biologically active form, and T4 acts in peripheral tissues by conversion of T4 to T3 by deiodinase (DIO)‐1 and DIO‐2. To some extent, the TH levels determine the pathological development and outcome of GD. Antithyroid drugs (ATDs) are primarily used as a treatment for GD to inhibit TH synthesis. However, after the recommended course of treatment, more than half of the patients with GD experience a relapse of hyperthyroidism after drug withdrawal.^3^, ^4^ Interestingly, high levels of THs are an independent risk factor for the recurrence of hyperthyroidism,^5^, ^6^ while recurrent hypothyroidism in patients on medication may reduce the recurrence of Graves' hyperthyroidism (GH) after drug withdrawal.^7^ Furthermore, long‐term control of TH levels with low‐dose ATDs also significantly reduced the recurrence rate of GD.^8^

Thyroid hormones are stress‐sensitive, with their levels susceptible to external environmental influences; thus, because of the crosstalk between THs and the immune system, elevated TH levels should be regarded as an immune regulator in the development of GD. B‐cell‐activating factor (BAFF) has been found to be aberrantly expressed in a variety of autoimmune diseases and is essential for regulating B cell differentiation, immunoglobulin type switching, and antibody production.^9^, ^10^, ^11^, ^12^, ^13^ Overexpression of BAFF in the peripheral blood of patients with GD has been found, and the frequency of CD11c+ autoreactive secretory B cells (ASCs), which may be a potential therapeutic target, was positively correlated with TRAb in the serum of patients with GD.^14^, ^15^ However, the results of studies on how THs affect immune cell function remain controversial because of the lack of a comprehensive understanding of the local role of autoimmune cell functions. Additionally, although TRAb is usually considered the cause of persistently elevated T3 in GD, there are few studies on the inverse effects of high‐circulating TH levels on the abnormal differentiation of local ASCs in the thyroid and the production of TRAb in patients with GD.

In the present study, the important bridging role of BAFF overexpression—stimulated by high TH levels—in inducing differentiation of ASCs was clarified in vivo and in vitro, which provides novel evidence for the interaction of THs with the immune system in the pathogenesis of GD.

## 2 PATIENTS AND METHODS

### 2.1 Participants

Eighteen patients with GD and 11 healthy donors were recruited for this study from the Nanjing First Hospital. All patients satisfied the diagnostic criteria for GD—clinically and biochemically verified hyperthyroidism, a positive TRAb, and 12–18 months or more with the recommended treatment course of ATDs from the diagnosis of GD.^16^ Ten patients with clinical remission after ATD treatment and whose thyroid function suggested elevated free T3 (FT3) and free thyroxine (FT4) levels 3 months after drug withdrawal, were categorized into the GH recurrence (GHR) group; eight patients with matched values within the normal range were categorized into the GH non‐recurrence (GHNR) group (satisfied GD criteria before their initial drug treatment). Clinical evaluation of the patients included a review of patient history, physical examination, and thyroid ultrasonography. Laboratory and diagnostic tests included the determination of serum FT4, FT3, sensitive thyroid‐stimulating hormone (s‐TSH), and TRAb levels. Patients with a low number of leukocytes (fewer than 3.5 × 10^9^), subacute thyroiditis, hyperfunctioning thyroid nodules, iodine hyperthyroidism, drug‐induced hyperthyroidism, or other causes of hyperthyroidism were all excluded from the study. Additionally, participants with other autoimmune diseases, acute or chronic infections, co‐occurring systemic diseases, or cancers were strictly excluded.

Surgical and peripheral blood samples were obtained from 12 patients who had undergone surgery for benign thyroid nodules. Benign thyroid nodules were diagnosed using thyroid ultrasonography, computed tomography, and technetium‐99 radioisotope scans, and the patients did not receive any specific treatment for thyroid diseases. In addition, the levels of THs and autoantibodies were within the normal ranges (Table 2). A normal specimen near the thyroid nodule was obtained during surgery, and the diagnosis was confirmed postoperatively via pathological examination.

Written informed consent was obtained from the participants prior to the study, and ethical permission was obtained for the use of their specimens. This study was approved by the local ethics committee of Nanjing First Hospital (No.: DWSY‐2000629).

### 2.2 Thyroid function assay

Serum TSH, FT3, FT4, TGAb, TPOAb, and TRAb levels in patients with GD were measured using chemiluminescence at the Nuclear Medicine Center of Nanjing First Hospital, according to the manufacturer's instructions.

### 2.3 Cell isolation and collection

Thyroid follicular cells were prepared as previously described.^17^, ^18^ Thyroid tissues were cut and digested with 250 IU/ml collagenase II and 0.25% trypsin (Gibco) in D‐Hank's solution for 90 min. After washing with D‐Hank's solution, the cell suspension was washed and filtered through a 125 μm strainer, and the suspension was placed in a medium containing 10% fetal bovine serum (FBS), 2 mM glutamine, and 50 μg/ml penicillin/streptomycin at 37°C and 5% CO_2_. Tissue mononuclear cells (MCs) and peripheral blood mononuclear cells (PBMCs) were collected using Ficoll–Hypaque density gradient centrifugation (LTS1077, TBD Science) and washed twice with phosphate‐buffered saline. CD19+ B cells were isolated from MCs using CD19+ B cell isolation kits (130‐052‐201, Miltenyi Biotec) according to the manufacturer's protocol.

### 2.4 T3 treatment in vitro

Isolated cells from thyroid tissues were cultured with T3 (CAS 5817‐39‐0, Meilun Biotech) at final concentrations of 10^3^, 10^4^, 10^5^, and 10^6^ pmol/L in 1640 RPMI medium with 10% FBS (Thermo Fisher Scientific) for 72 h in a 5% CO_2_ incubator at 37°C, as previously described.^19^

### 2.5 RNA interference

The BAFF siRNA sequence was 3′‐CGGGAGAATGCACAGATTT‐5′. The non‐target control sequence was 5′‐TTCTCCGAACGTGTCACGT‐3′ (GeneChem). Transfection was performed using Lipofectamine 2000 (Invitrogen), and the knockdown efficiency was verified using quantitative PCR or western blotting.

### 2.6 Enzyme‐linked immunosorbent assay

BAFF levels in the supernatant and serum samples were analyzed using a human BAFF (SEB686Hu, Cloud‐Clone Corp) ELISA kit, which was operated strictly according to the manufacturer's protocol.

### 2.7 Western blotting

Total cells were homogenized, and total proteins were extracted using a protein extraction kit (KGP902, KeyGen Biotech). Equal amounts of protein were separated via 15% SDS‐PAGE and transferred to polyvinylidene fluoride membranes. Subsequently, the membranes were blocked with 5% non‐fat milk for 2 h and incubated with anti‐BAFF (ab16081, Abcam) and anti‐β‐actin (ab8227, Abcam) antibodies at 4°C overnight. The membranes were washed thrice with TBST buffer and incubated with horseradish peroxidase‐conjugated secondary antibodies. Detection was visualized using an enhanced chemiluminescence system.

### 2.8 Quantitative reverse transcription polymerase chain reaction

Total RNA was extracted from MCs using the conventional TRIzol reagent (Life Technologies). Reverse transcription was performed using the PrimeScript™ RT Reagent Kit (Takara Bio Inc.) with gDNA Eraser, in accordance with the manufacturer's instructions for the two‐step quantitative reverse transcription polymerase chain reaction. The normalized expression values for each transcript were calculated as the quantity of target gene mRNA relative to the quantity of β‐actin mRNA, using the $2^{-\Delta \Delta C_{t}}$ method. The forward and reverse primers were as follows (5′–3′): BAFF, GAGCAATCCAATCGGAGGGT and TTGATGTCCTGCGTGCACTA; β‐actin, TCAAGATCATTGCTCCTCCTGAG and ACATCTGCTGGAAGG TGGACA.

### 2.9 Flow cytometry

The cell suspensions were subjected to flow cytometry analysis. Human blood cells were incubated with PE‐A‐anti‐CD11c, FITC‐anti‐CD19, PerCP‐cy5.5‐anti‐CD19, FITC‐anti‐CD11c, and PE‐A‐anti‐IgG (Cat. Nos.: 552761, 521042, 555435, 565018, and 301434, respectively; BD Biosciences). The samples were acquired using a BD Canton‐II flow cytometer.

### 2.10 Statistical analysis

All statistical tests were performed using the GraphPad Prism V.8.0 software. To examine the significance of mean differences between groups, we used two‐tailed paired or unpaired Student's *t* tests for normally distributed data. The correlation between the two variables was determined using the Pearson's test. All *p*‐values were two‐sided, and statistical significance was set at *p* < 0.05.

## 3 RESULTS

### 3.1 Serum BAFF levels in patients with GD were significantly and positively correlated with FT3, FT4, and TRAb levels

**FIGURE 1:**
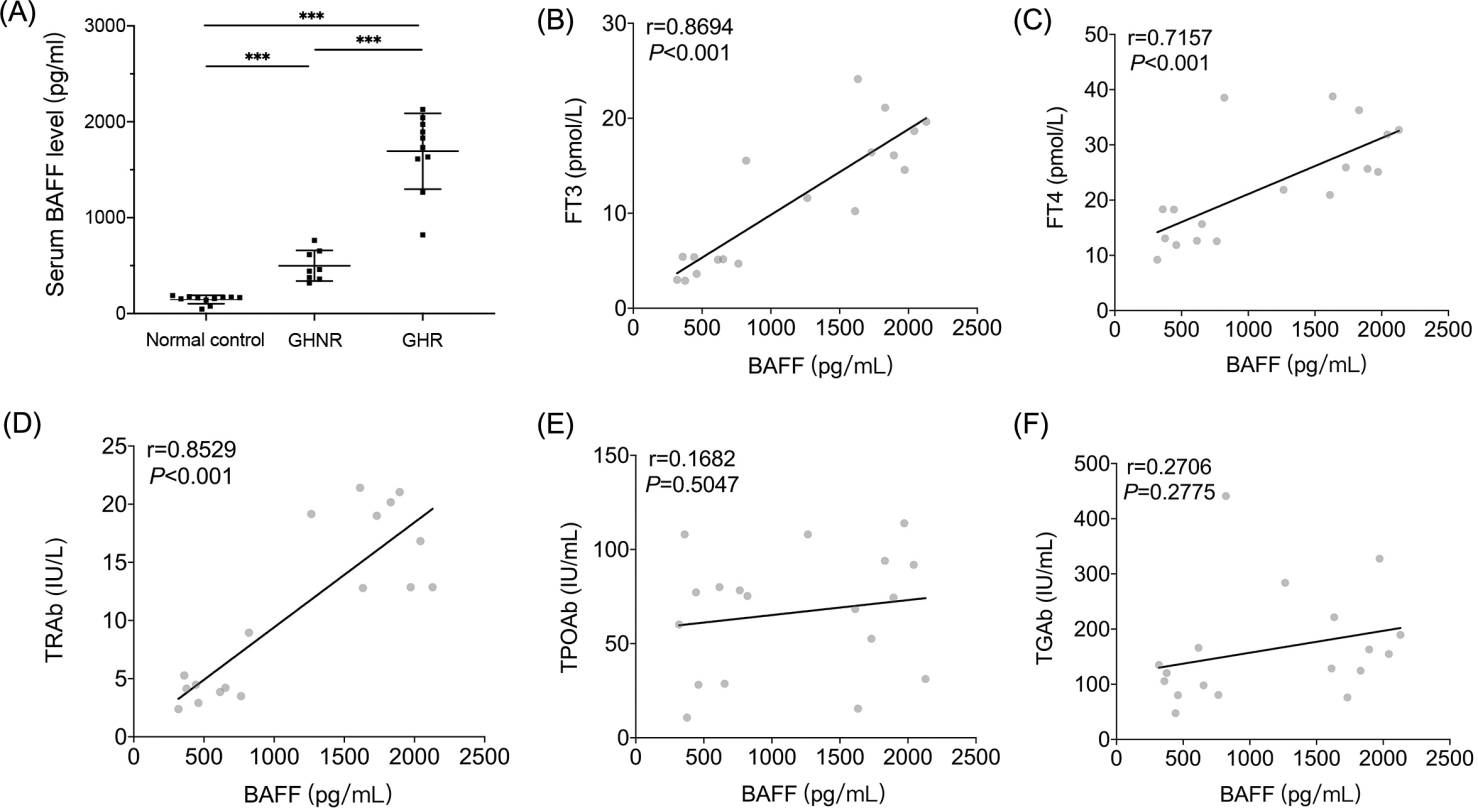
Serum BAFF levels were elevated in patients with GH and showed a significant positive correlation with FT3, FT4, and TRAb levels. (A) Two‐by‐two comparison of serum BAFF levels in the three groups enrolled. (B–F) Correlation analysis of serum BAFF levels with FT3, FT4, TRAb, TPOAb, and TGAb levels in all GD patients (both GHNR and GHR groups), respectively. Statistical significance is assessed by the two‐tailed unpaired Students' *t* test, and the correlation between the two variables was determined using the Pearman's test ***p < 0.001.

The clinical data of the three groups of participants were statistically analyzed. As expected, patients in the GHR group showed a significant increase in the levels of TRAb, FT3, and FT4 compared to those in the GHNR group (Table 1). Serum BAFF levels were significantly higher in both the GHR and GHNR groups of patients with GD compared to the control group, which is consistent with the results of previous studies.^20^, ^21^, ^22^ However, accompanied by increased levels of THs (FT3 and FT4) in the GHR group, the levels of serum BAFF were markedly higher than those in the GHNR group (Figure 1A). Pearson's correlation analysis further suggested that serum BAFF levels in patients with GD showed a significant positive correlation with FT3, FT4, and TRAb levels, and no correlation with TPOAb and TGAb levels (Figure 1B,C).

**TABLE 1:** Clinical characteristics in patients with GD and healthy donors

	Normal control	GHNR	GHR	Normal value
	(*n* = 11)	(*n* = 8)	(*n* = 10)	
Age (years)	41.30 ± 8.47	36.28 ± 14.15	41.08 ± 10.37	–
Gender (male/female)	3/8	2/6	3/7	–
TSH (mIU/L)	1.80 ± 0.42	0.66 ± 1.0^***a***^	0.48 ± 0.95^***b***^	0.45–4.94
FT3 (pmol/L)	3.80 ± 1.30	4.41 ± 1.07	16.80 ± 4.23^***b,c***^	2.63–5.70
FT4 (pmol/L)	13.80 ± 2.15	13.96 ± 3.22	29.77 ± 6.73^***b,c***^	9.00–19.0
TRAb (IU/L)	0.51 ± 0.28	3.84 ± 0.91^***a***^	16.51 ± 4.33^***b,c***^	<1.75
TPOAb (IU/mL)	12.51 ± 1.88	58.91 ± 33.26^***a***^	72.51 ± 31.97^***b***^	<34
TGAb (IU/mL)	13.91 ± 2.17	104.20 ± 36.66^***a***^	211.1 ± 110.7^***b,c***^	<115
WBC (10^9^/L)	5.32 ± 1.04	4.82 ± 1.25	4.40 ± 1.53	3.5–9.5
ALT (U/L)	22.40 ± 4.33	44.65 ± 13.62^***a***^	47.90 ± 11.80^***b***^	9–50
AST (U/L)	18.77 ± 3.70	28.77 ± 8.79^***a***^	24.61 ± 10.01^***b***^	15–40

*Note*: Clinical data are presented as *X* ± SD. ^a,b^Reflects significant differences compared with the healthy donors (normal control), while, ^c^represents comparisons with the GHNR group. Statistical significance is denoted as *p* < 0.05 assessed by the two‐tailed unpaired Students' *t* test.

Abbreviations: ALT, alanine transaminase; AST, aspartate aminotransferase; FT3, free triiodothyronine; FT4, free thyroxine; TGAb, thyroglobulin antibody; TPOAb, thyroid peroxidase; TRAb, thyrotropin receptor antibody; TSH, thyroid‐stimulating hormone; WBC, the actual number of white blood cells.

**TABLE 2:** Clinical characteristics in patients with benign thyroid nodules

	*X* ± SD	Range	Normal value
Age (years)	45.31 ± 10.95	29–60	–
Gender(male/female)	4/8	–	–
TSH (mIU/L)	1.78 ± 0.62	0.67–2.73	0.45–4.94
FT3 (pmol/L)	4.32 ± 0.62	3.48–5.62	2.63–5.70
FT4 (pmol/L)	15.73 ± 0.62	14.30–16.34	9.00–19.0
TRAb (IU/L)	0.42 ± 0.10	0.21–0.56	<1.75
TPOAb (IU/ml)	10.67 ± 5.65	2.72–20.56	<34
TGAb (IU/ml)	33.65 ± 12.74	10.46–56.14	<115
WBC (10^9^/L)	5.71 ± 1.2	4.06–8.14	3.5–9.5
ALT (U/L)	19.35 ± 7.11	6.54–27.97	9–50
AST (U/L)	21.60 ± 6.77	10.05–30.07	15–40

*Note*: Clinical data are presented as *X* ± SD.

Abbreviations: ALT, alanine transaminase; AST, aspartate aminotransferase; FT3, free triiodothyronine; FT4, free thyroxine; TGAb, thyroglobulin antibody; TPOAb, thyroid peroxidase; TRAb, thyrotropin receptor antibody; TSH, thyroid‐stimulating hormone; WBC, the actual number of white blood cells.

### 3.2 Abnormally differentiated CD11c+ autoreactive B cells were positively correlated with BAFF and TRAb levels in GH

**FIGURE 2:**
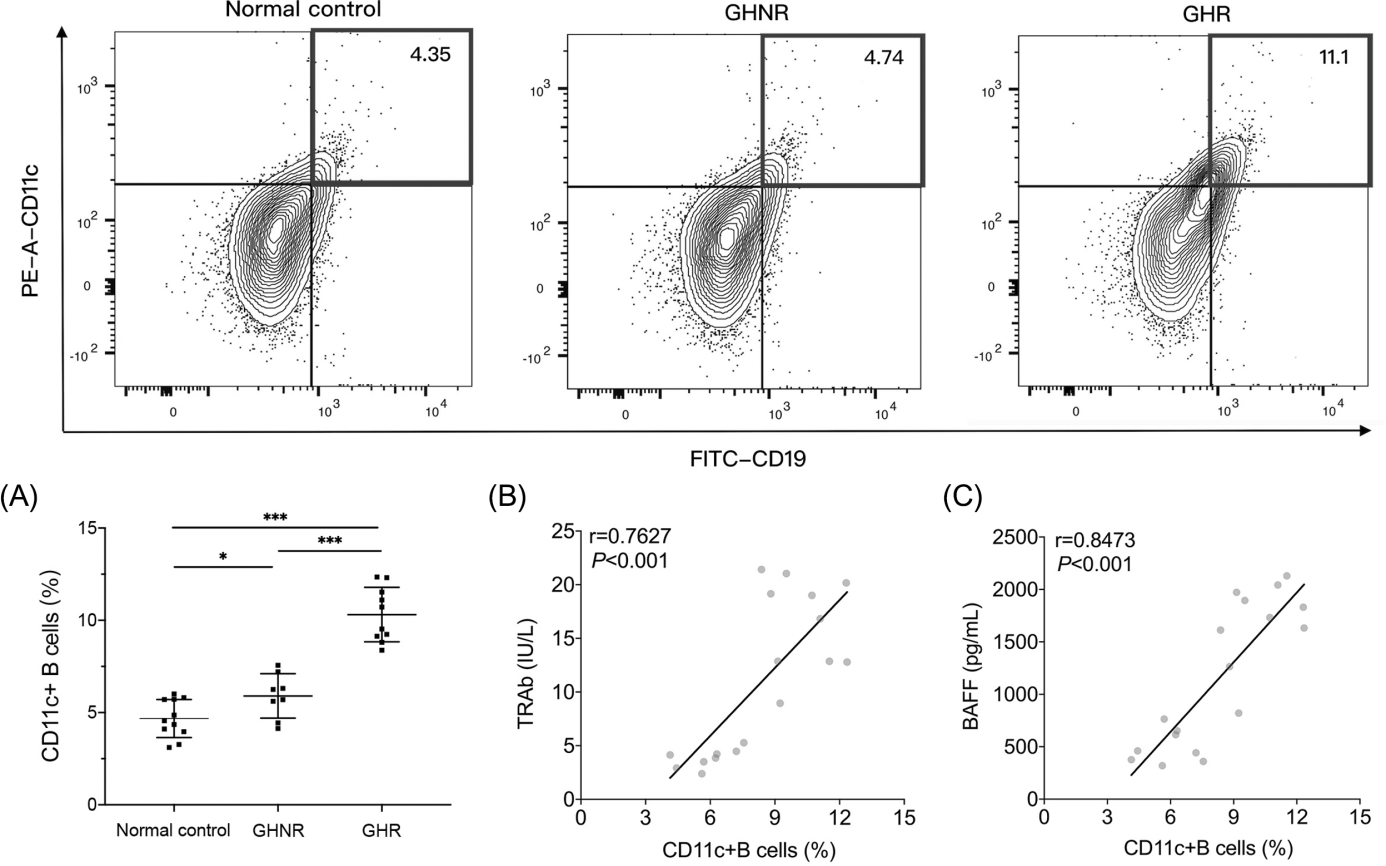
CD11c+ autoreactive B cells were elevated in PBMCs of patients with GH and showed a significant positive correlation with BAFF and TRAb levels. (A) Two‐by‐two comparison of CD19+CD11c+ cells ratio in the three groups enrolled. (B, C) Correlation analysis of CD19+CD11c+ cells ratio with TRAb and BAFF levels in all GD patients (both GHNR and GHR groups), respectively. Statistical significance is assessed by the two‐tailed unpaired Students' *t* test, and the correlation between the two variables was determined using the Pearman's test. *p < 0.05, ***p < 0.001

Peripheral blood mononuclear cells from the three groups were isolated, and the proportion of CD11c+CD19+ B cells was examined. Although the proportion of CD11c+ autoreactive B cells was still elevated in the GHNR group compared to that in the normal control group, the proportion of CD11c+ autoreactive B cells was significantly lower than that in the GHR group (Figure 2A), implying that CD11c+ autoreactive B cells are abnormally differentiated in patients having GD with GH recurrence. Correlation analysis also indicated that the proportion of CD11c+ autoreactive B cells in the peripheral blood of patients with GD positively correlated significantly with TRAb and BAFF levels (Figure 2B, C).

### 3.3 Elevated BAFF levels in cultured human TFC supernatants stimulated by high levels of T3

**FIGURE 3:**
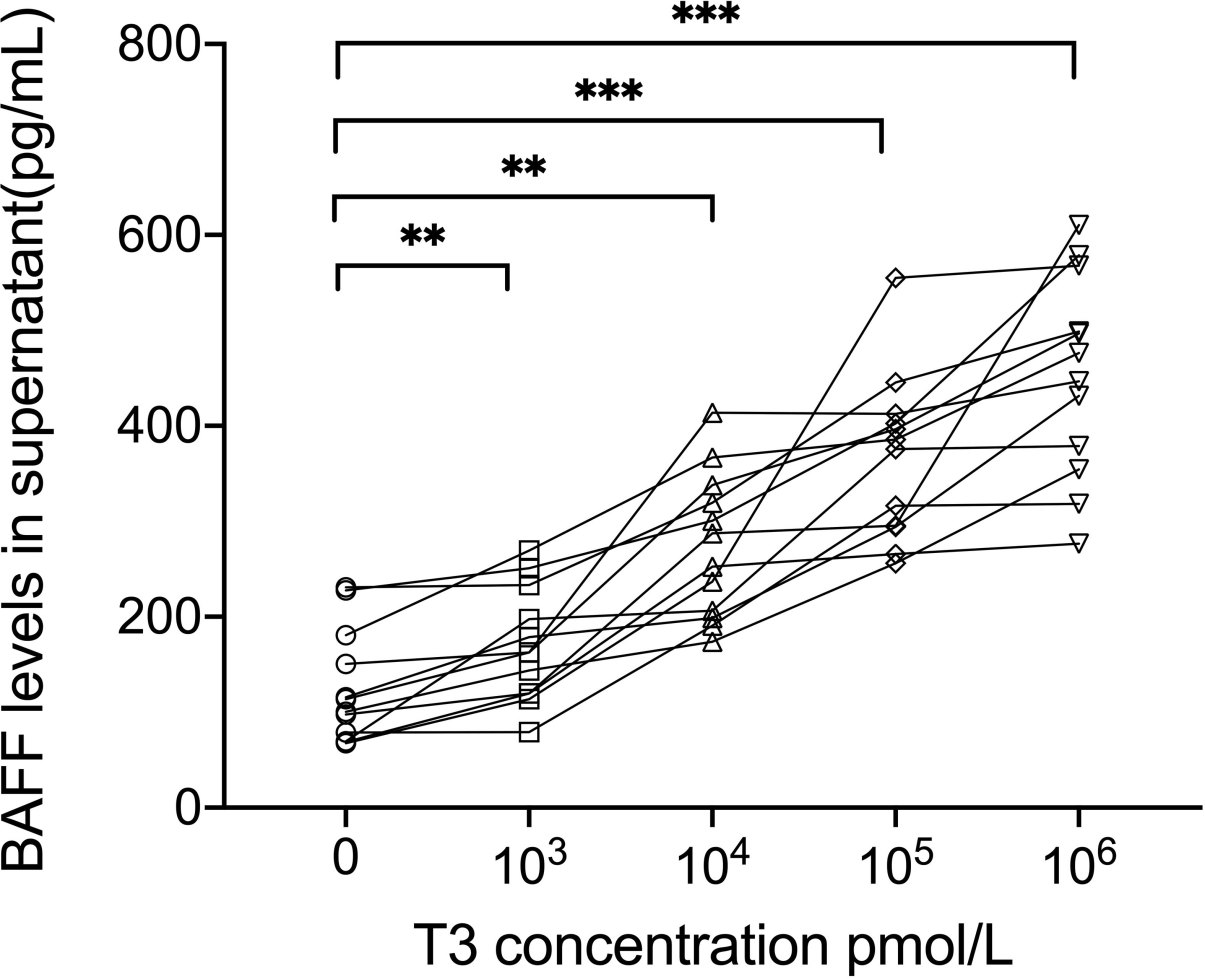
The BAFF levels in supernatant of TFCs treated with various concentrations of T3 stimulation for 72 h. Comparison between the BAFF levels and that of paired BAFF levels in each sample (*n* = 12 samples per group). Statistical significance is assessed by the two‐tailed paired Students' *t* test (*n* = 3 independent biological experiments); 0 pmol/L group: 125.1 ± 59.3 pg/ml, 10^3^ pmol/L group: 169.3 ± 59.20 pg/ml, 10^4^ pmol/L group: 273.8 ± 76.43 pg/ml, 10^5^ pmol/L group: 366.8 ± 86.15 pg/ml, 10^6^ pmol/L group: 453.0 ± 105.8 pg/ml. **p < 0.01; ***p < 0.001

Considering that T3, the main bioactive TH, is three to four times more potent than T4,^23^ T3 administration avoids the need for intracellular conversion of T4 to T3, which, as mentioned earlier, requires most of the action of T4 to occur through enzymatic deiodination.^24^ In our study, we used different concentrations of T3, rather than T4, to simulate high‐circulating TH levels in vitro. The results showed that the BAFF levels in the supernatant of the TFCs of each sample progressively increased with elevated T3 levels (10^4^, 10^5^, and 10^6^ pmol/L) (Figure 3).

### 3.4 High levels of T3‐induced overexpression of BAFF in MCs of the thyroid

**FIGURE 4:**
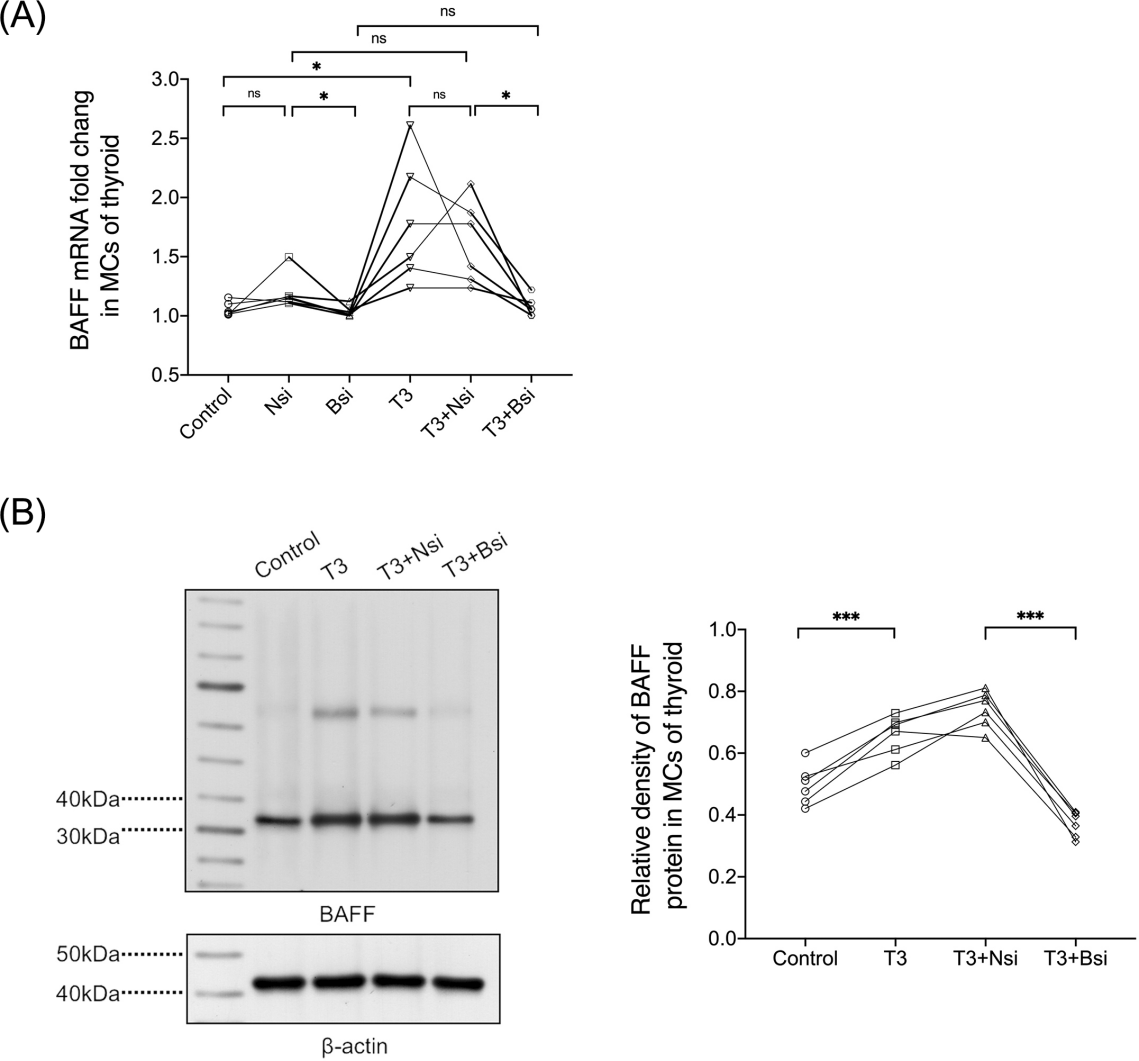
High levels of T3‐induced overexpression of BAFF in MCs of human thyroid. (A) Relative expression of BAFF mRNA in MCs of thyroid after four different interventions in the control (0 pmol/L) group, no‐target control siRNA group (Nsi), BAFF siRNA group (Bsi), T3 (10^6^ pmol/L) group, T3 (10^6^ pmol/L) + no‐target control siRNA group (T3 + Nsi), and T3 (10^6^ pmol/L) + BAFF siRNA group (T3 + Bsi), respectively (*n* = 6 samples per group). Control group: 1.06 ± 0.06, Nsi group: 1.20 ± 0.15, Bsi group: 1.04 ± 0.05, T3 group: 1.78 ± 0.52, T3 + Nsi group: 1.62 ± 0.35, T3 + Bsi group: 1.08 ± 0.08.*p < 0.05. (B) Comparative expression of BAFF protein in MCs of thyroid after four different interventions in control group (0 pmol/L), T3 group (10^6^ pmol/L), T3 (10^6^ pmol/L) +Nsi group and T3 (10^6^ pmol/L) +Bsi group, respectively (*n* = 6 samples per group). Control group: 0.49 ± 0.06, T3 group: 0.65 ± 0.05, T3+Nsi group: 0.71 ± 0.06, T3+Bsi group: 0.40 ± 0.05. ***p < 0.001. Statistical significance is assessed by two‐tailed paired Students’ *t* test. (*n* = 3 independent biological experiments).

BAFF, a member of the tumor necrosis factor family, is mainly expressed by immune cells such as monocytes, macrophages, neutrophilic granulocytes, and dendritic cells.^25^ Thus, equal amounts of thyroid MCs from each sample were randomly divided into control (0 pmol/L), no‐target control siRNA (Nsi), BAFF siRNA (Bsi), T3 (10^6^ pmol/L), T3 (10^6^ pmol/L) + no‐target control siRNA (T3 + Nsi), and T3 (10^6^ pmol/L) + BAFF siRNA (T3 + Bsi) groups for co‐culture. The knockdown effect of BAFF siRNA was verified; BAFF mRNA in MCs showed a decrease in the Bsi group compared to the Nsi group after 72 h of incubation without T3 stimulation, and the interference of BAFF siRNA and no‐target siRNA on the results was excluded (Figure 4A). In addition, the expression of BAFF protein was elevated in the T3 group compared to the control group, and, after precise knockdown of BAFF mRNA expression, BAFF protein at the same high T3 level was decreased in the T3 + Bsi group compared to the T3 + Nsi group, which is consistent with the results of BAFF mRNA (Figure 4B).

### 3.5 Overexpression of BAFF caused abnormal differentiation of CD11c+IgG+ B cells

**FIGURE 5:**
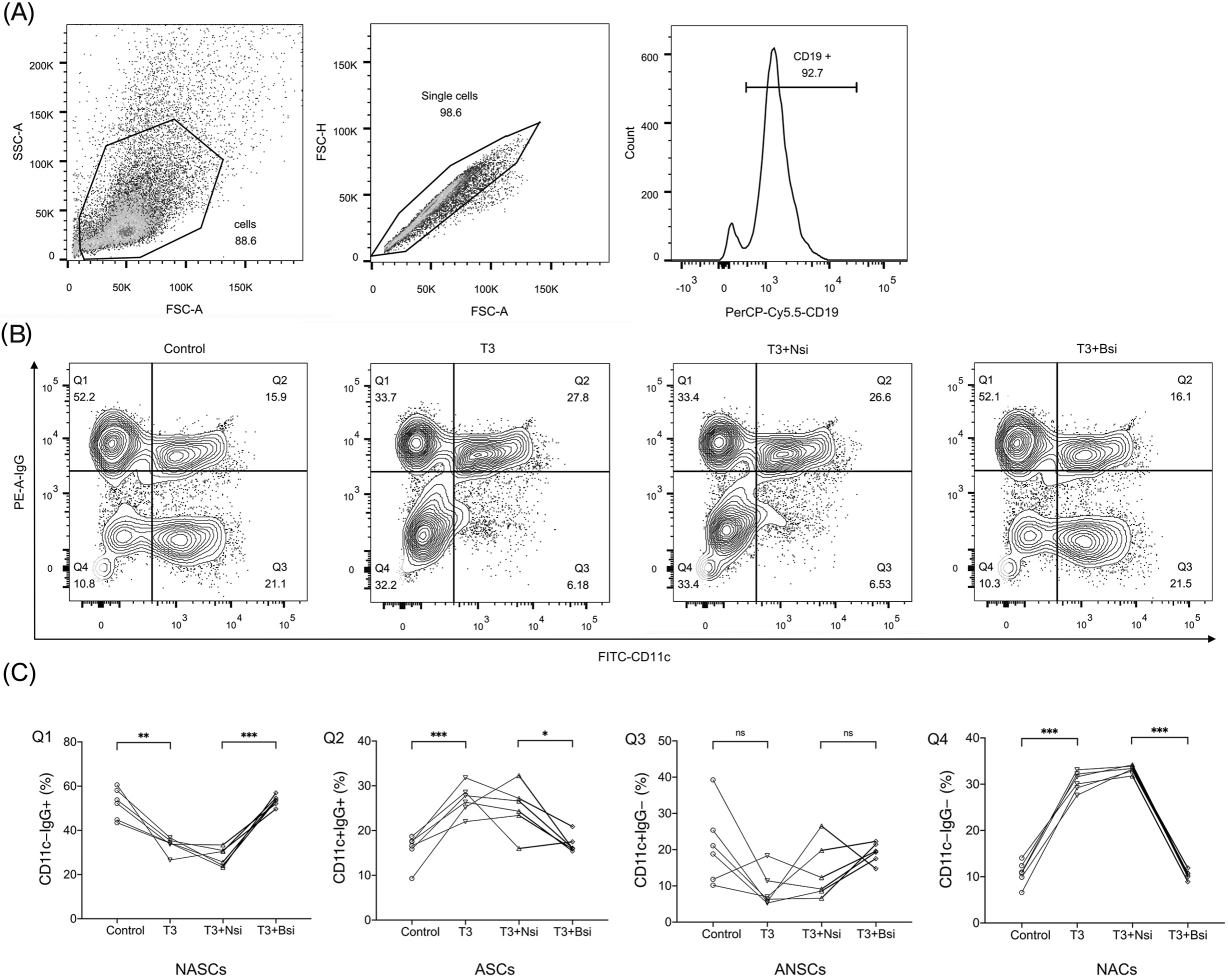
Overexpression of BAFF caused increase in proportion of CD11c+IgG+B cells. (A) Flow cytometry assays for the purity of CD19+ B cells each sample >90% isolated by magnetic beads. (B) CD11c and IgG gating on the B cells of the control, T3, T3+Nsi, and T3+Bsi groups, respectively. (C) Comparison of the proportion of CD11c‐IgG+ (NASCs), CD11c+IgG+ (ASCs), CD11c+IgG‐ (ANSCs), and CD11c‐IgG‐ (NACs) in B cells between the control, T3, T3 + Nsi, and T3 + Bsi groups (*n* = 6 samples per group). Q1: Control, 52.2 ± 6.9, T3, 33.5 ± 3.5, T3 + Nsi, 28.0 ± 4.2, T3 + Bsi, 53.5 ± 2.5; Q2: Control, 16.0 ± 3.4, T3, 27.0 ± 3.3, T3 + Nsi, 25.0 ± 5.4, T3 + Bsi, 17.0 ± 2.1; Q3: Control, 21.1 ± 10.6, T3, 9.0 ± 5.1, T3 + Nsi, 13.8 ± 7.8, T3 + Bsi, 19.2 ± 2.7; Q4: Control, 10.8 ± 2.5, T3, 30.6 ± 2.1, T3 + Nsi, 33.3 ± 0.8, T3 + Bsi, 10.4 ± 1.0. ns, *p* > 0.05,*p < 0.05, ** *p* < 0.01, ***p < 0.001, Statistical significance is assessed by two‐tailed paired Students’ *t* test. (*n* = 3 independent biological experiments).

A growing body of evidence suggests that CD11c+ B cells, which are autoreactive B cells, have been observed to expand in various autoimmune diseases.^26^, ^27^, ^28^, ^29^, ^30^ However, the effect of T3 on the activation and differentiation of autoreactive B cells remains unknown and has been poorly studied in GD. To further observe the role of BAFF overexpression, the CD19+ B cells of thyroid isolated and purified using magnetic beads were labeled with the corresponding antibodies (Figure 5A) and classified into CD11c–IgG+ B cells (non‐autoreactive secretory antibody cells, NASCs), CD11c+IgG+ B cells (autoreactive antibody‐secretory cells, ASCs), CD11c+IgG– B cells (autoreactive non‐secretory cells, ANSCs), and CD11c–IgG– B cells (non‐autoreactive B cells, NACs) (Figure 5B). The results showed that the proportion of ASCs was higher in the T3 group than in the control group, whereas knockdown of BAFF expression resulted in a significant decrease in the proportion of ASCs in the T3+Bsi group compared to that in the T3 + Nsi group. This is consistent with the results for NACs and is opposite to those for NASCs. Furthermore, none of the ANSCs proportions changed significantly (Figure 5C).

## 4 DISCUSSION

Although many patients with GD and the recurrence of hyperthyroidism have demonstrated substantial unmet clinical needs, conventional therapies, including ATDs, radioactive iodine, and surgery, have remained mostly unchanged over the past few decades. New treatment options should be combined with a deeper understanding of the autoimmune pathogenesis of GD.^31^, ^32^, ^33^, ^34^

The interaction between THs and the immune system involves a bidirectional crosstalk. On the one hand, autoimmune‐related pathologies can affect this balance. On the other hand, THs regulate innate and adaptive immune responses, as immune cells are the direct target cells of THs.^35^, ^36^, ^37^ In patients with GD, we found for the first time that overexpression of BAFF caused abnormal differentiation of ASCs and was positively correlated with FT3, FT4, and TRAb levels. In addition, the fact that BAFF overexpression is also present in the GHNR group needs to be elucidated, which may explain the initial development of GD, due to TRAb being the cause of GH. Since the results of our correlation analysis were for all patients with GD enrolled including both GHR and GHNR groups, and did not assess patients with a primary diagnosis of GD, which is a shortcoming of our study. Based on the knowledge that the activity and regulation of T4 on B cells is not clear, we selected T3 as a representative TH that plays a major biological role in vitro.^24^ We observed that elevated BAFF expression levels in TFCs and MCs stimulated by high concentrations of T3 in vitro may play a key role in the differentiation of B cells into ASCs, which promotes the survival and maturation of low‐affinity self‐reactive transitional B cells.^21^, ^38^, ^39^ In addition, we also found overexpression of BAFF in the thyroid of an established high‐circulating T3 mouse model (for more details, see Figure S1). It is worth noting that the proportion of CD11c+ autoreactive B cells was positively correlated with TRAb, suggesting that ASCs may participate in the development of GD through the secretion of autoantibodies, which in turn leads to the recurrence of GH.^14^

As reported, CD11c+ B cells also contribute to the pathogenesis of GD in multiple ways through the secretion of pro‐inflammatory cytokines (IL‐1β, IL‐6, IL‐17A, IFN‐γ, and IL‐9) and chemokines (IL‐8, CXCL10, RANTES, MIP‐1α/β, and MCP‐1).^40^, ^41^ Based on the knowledge that CD11c+ B cells infiltrate the thyroid of patients with GD with a phenotype similar to that of CD11c+B cells in peripheral blood,^14^ we further identified BAFF as a key factor causing differentiation of CD11c+IgG+ ASC subsets induced by high levels of T3, which are highly expressed in unswitched memory B cells, switched memory B cells, early memory mature B cells, germinal center B cells, plasma blasts, and plasma cells in the thyroid.^14^ We found that in CD11c+ autoreactive B cells, T3 prompted the differentiation of B cells toward IgG+ B cells via BAFF, whereas in NACs, high T3 prompted the differentiation of such B cells toward IgG– B cells via BAFF, suggesting that the production effect of ASCs may be higher. The lack of further evaluation of CD38 and CD27 markers regarding the differentiation of plasma cells and memory cells and in vivo verification needs to be improved. Further research with an expanded sample size and a comprehensive understanding of the interaction between other innate and adaptive immune cells in the thyroid is needed.

In conclusion, BAFF should be further evaluated as a checkpoint for predicting GH emergence and relapse. However, our study demonstrates a critical bridging role for BAFF in high T3‐induced ASCs and provides novel insights into a clinical response to complement the potential targets of therapeutic immunosuppression against recurrences of GD, in addition to controlling T3 levels through conventional therapy.

## AUTHOR CONTRIBUTIONS

Xiao‐Ming Mao conceived the study and designed experiments. Shu Liu and Jing‐Jing Miao designed and performed experiments, analyzed the data, and wrote the article. Xiao Zhou provided critical feedback during the preparation of the article. Qi Sun provided assistance with experiments. All authors read, edited, and approved the article.

## FUNDING INFORMATION

This work was supported by the National Natural Science Foundation of China (81570710).

## CONFLICT OF INTEREST

All the authors have nothing to disclose.

## Supporting information


Appendix S1

## Data Availability

The data that support the findings of this study are available from the corresponding author upon reasonable request. The data are not publicly available due to privacy or ethical restrictions.
