# Supplementary material for: High levels of thyroid hormones promote recurrence of Graves' disease via overexpression of B‐cell‐activating factor
Source: J Clin Lab Anal. 2022 Sep 13;36(10):e24701. doi: 10.1002/jcla.24701 (PMC9550970; doi:10.1002/jcla.24701)
Supplement: Supplementary file 1 — Appendix S1 [file JCLA-36-e24701-s001.docx]

**Supplementary**

**Methods**

**Animal experimental design and model preparation**

C57BL/6 mice, aged 6-8 weeks and weighing 12-22 g, were randomly and equally divided into the control, T3, T3+negative control shRNA (T3+NCs), and T3+BAFF shRNA (T3+Bs) groups (n=8 per group). The tail veins of the mice in the T3+Bs and T3+NCs groups were intravenously injected with BAFF shRNA or negative control, respectively. Four weeks later, the T3, T3+NCs, and T3+Bs groups were injected subcutaneously with T3 (5 μg/10 g) (M0818A, Meilun Biotech) every day for 6 weeks, while the control group was subcutaneously injected with the same volume of saline. The study was approved by the Experimental Animal Ethics Committee of Nanjing Medical University and the experimental procedures were in accordance with the Guide for the Application of Laboratory Animals.

**Lentivirus production and in vivo infection**

Lentivirus construction encompassing BAFF was generated using the AdMax (Microbix) and pSilencer™ adeno 1.0-CMV (Ambion) systems. The viruses were packaged, amplified in 293T cells, and purified. Titering was performed on 293T cells using the Adeno-X Rapid Titer kit. For in vivo infection, lentiviruses were suspended in 100 µL of PBS, containing 7.6 × 10^7^ IFUs of loaded lentivirus per mouse, and intravenously injected via the tail, as described previously^1^.

**Measurement of serum T3**

T3 levels in serum of mice were analyzed by using a T3 (CEA453Ge, Cloud-Clone Corp) enzyme-linked immunosorbent assay kit, according to the manufacturer’s instructions.

**Immunohistochemical staining (IHC)**

Specimens of thyroid were preserved in 10% formalin, dehydrated, and embedded in paraffin following routine methods. Paraffin sections were routinely dewaxed, incubated at room temperature with 3% H_2_O_2_, and repaired with 0.01 mol/L citric acid solution. Anti-BAFF antibody (ab16081, Abcam) was used for immunohistochemical staining, and the DAB-H_2_O_2_ colorant was added for color rendering. Hematoxylin was used for dehydration and sealed with neutral gum for observation under a light microscope. The cytoplasm of BAFF-positive cells was brownish yellow, and cells were detected using a pathological image analysis system. Results of Area% were statistically analysed by Image J software.

**Statistical analysis**

Statistical tests were performed using GraphPad Prism software. Two-tailed unpaired Student’s t-tests for normally distributed data. Statistical significance was set at *P* < 0.05.

**References**

1. Wang J, Guo T, Peng Q-S, Yue S-W, Wang S-X. Berberine via suppression of transient receptor potential vanilloid 4 channel improves vascular stiffness in mice. *J Cell Mol Med.* 2015;19(11):2607-2616.

**Supplementary legend**


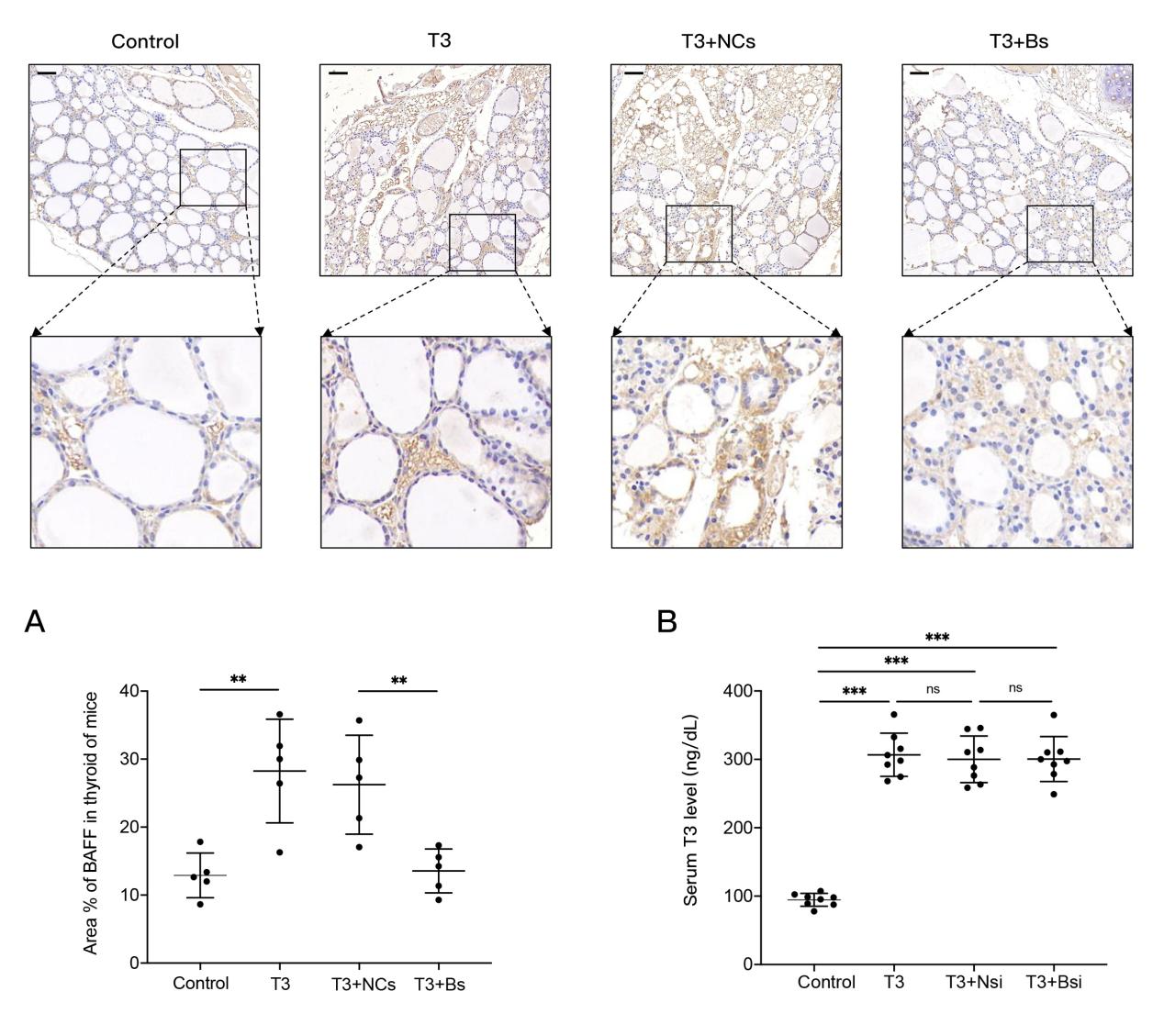


FigueS1. BAFF is significantly overexpressed in the thyroid tissue of a high circulating T3 mouse model（A）Comparison of IHC staining of BAFF in the thyroid gland of the control, T3, T3+NCs and T3+Bs groups of mice. n=5 random views were selected. Sar bar=50μm. (B) Two-by-two comparison of peripheral blood serum T3 in four groups of mice above. * *P*<0.05, ^**^ *P* <0.01; ^***^ *P*<0.001.Statistical significance is assessed by two-tailed unpaired Students’ t-test. (n = 3 independent biological experiments).
